# New Downstream Signaling Branches of the Mitogen-Activated Protein Kinase Cascades Identified in the Insect Pathogenic and Plant Symbiotic Fungus *Metarhizium robertsii*

**DOI:** 10.3389/ffunb.2022.911366

**Published:** 2022-05-30

**Authors:** Dan Tang, Xingyuan Tang, Weiguo Fang

**Affiliations:** MOE Key Laboratory of Biosystems Homeostasis & Protection, College of Life Science, Institute of Microbiology, Zhejiang University, Hangzhou, China

**Keywords:** *Metarhizium*, MAPK cascade, entomopathogenicity, conidiation, signaling pathway

## Abstract

Fungi rely on major signaling pathways such as the MAPK (Mitogen-Activated Protein Kinase) signaling pathways to regulate their responses to fluctuating environmental conditions, which is vital for fungi to persist in the environment. The cosmopolitan *Metarhizium* fungi have multiple lifestyles and remarkable stress tolerance. Some species, especially *M. robertsii*, are emerging models for investigating the mechanisms underlying ecological adaptation in fungi. Here we review recently identified new downstream branches of the MAPK cascades in *M. robertsii*, which controls asexual production (conidiation), insect infection and selection of carbon and nitrogen nutrients. The Myb transcription factor RNS1 appears to be a central regulator that channels information from the Fus3- and Slt2-MAPK cascade to activate insect infection and conidiation, respectively. Another hub regulator is the transcription factor AFTF1 that transduces signals from the Fus3-MAPK and the membrane protein Mr-OPY2 for optimal formation of the infection structures on the host cuticle. Homologs of these newly identified regulators are found in other *Metarhizium* species and many non-*Metarhizium* fungi, indicating that these new downstream signaling branches of the MAPK cascades could be widespread.

## Introduction

*Metarhizium* is a genus in the Clavicipitaceae family, and it represents a continuum of species and strains that are extraordinarily versatile (St Leger and Wang, 2020). Some *Metarhizium* species have multiple lifestyles and they are pathogens, but most are also saprophytes, rhizosphere colonizers and beneficial root endophytes, with the ability to switch between these different lifestyles (Moonjely and Bidochka, 2019). Although *M. viride* and *M. granulomatis* are pathogenic to reptiles, most *Metarhizium* species are entomopathogenic, includin*g M. anisopliae, M. robertsii, M. brunneum, M. globosum, M. acridum, M. majus, M. flavoviride, M. frigidum, M. rileyi, M. pingshaense, M. lepidiotae*, and *M. guizhouense* (St Leger and Wang, 2020). The Nobel Prize winner Elie Metchnickoff initiated trials of a *Metarhizium* fungus for control of the wheat cockchafer *Anisoplia austriaca* in 1879 (Lord, 2005). Currently, *Metarhizium* fungi are being used worldwide as environmentally friendly alternatives to chemical insecticides to control agricultural and forest pests, and vectors of diseases (Zhao et al., 2016). Detailed mechanistic knowledge of *Metarhizium* pathogenicity to insects, conidiation and conidial tolerance to abiotic stresses is needed for mycoinsecticide improvement.

Insect infection by the *Metarhizium* fungi is initiated when conidia are attached to the insect cuticle and produce germ tubes that differentiate into infection structures called appressoria. The appressoria produce infection pegs, which penetrate the cuticle via a combination of mechanical pressure and cuticle degradation by diverse enzymes. In the insect hemocoel, the fungi undergo dimorphism from hyphae to yeast-like cells (i.e., blastospores), and the insect is killed by a combination of fungal growth and toxins. Finally, the mycelium grown in the hemocoel reemerges from the dead insect, resulting in a muscardine cadaver covered with dark green conidia. The formation of appressorium by *Metarhizium* is similar to plant pathogenic fungi such as *Magnaporthe oryzae* and *Colletotrichum lagenarium* (Kojima et al., 2002; Zhao et al., 2007). The protein kinase A and hydrophobins are functionally conserved during appressorial formation between *M. oryzae* and *Metarhizium* (Fang et al., 2009). *Metarhizium* fungi and mammal pathogenic fungi also have many parallels with regard to pathogenesis such as degrading proteinaceous integuments and evasion of the host innate immune system (Scully and Bidochka, 2006; Ortiz-Urquiza and Keyhani, 2013). Therefore, *Metarhizium* species can be used as representatives to study broad themes of fungal pathogenicity.

In the first dozen years of this century, robust genetic manipulation technologies including *Agrobacterium tumefaciens*-mediated fungal transformation and gene deletion based on homologous recombination had been developed for the *Metarhizium* fungi (Fang et al., 2006; Wang and St Leger, 2006; Xu et al., 2014; Su et al., 2018), and the genomes of *Metarhizium* species, *M. robertsii*, and *M. acridum*, were sequenced (Gao et al., 2011; Hu et al., 2014). This has been significantly speeding up the research on molecular mechanisms underlying insect infection and development by the *Metarhizium* fungi. Several groups in the field have been interested in regulators that control the insect infection and conidiation by *M. robertsii, M. acridum*, and *M. rileyi*. Many major regulators, which had been previously functionally characterized in other fungi including plant and mammal pathogenic fungi and even non-pathogenic fungi such as *Saccharomyces cerevisiae* and *Neurospora crassa*, were investigated in *Metarhizium*. These include a variety of transcription factors (Huang et al., 2015; Wei et al., 2017; Song et al., 2019), epigenetic regulators (Fan et al., 2017; Li et al., 2017; Wang et al., 2017; Lai et al., 2020), and several major signaling pathways such as cAMP-PKA, MAPK and GPCR (Fang et al., 2009; Jin et al., 2012, 2014; Liu et al., 2012; Chen et al., 2016; Song et al., 2016; Wang et al., 2018; Shang et al., 2021; Yu et al., 2021). However, these imitative studies recently revealed several new regulators of insect infection and conidiation by *M. robertsii*, which have not been functionally characterized in other fungi (Guo et al., 2017; Meng et al., 2021a; Zhang et al., 2021). Most noticeably, several new components constitute new downstream signaling branches of the MAPK signaling pathways in *M. robertsii*, which will be reviewed in this minireview.

## Mapk Cascades in *Metarhizium* Fungi

The evolutionarily conserved MAPK cascades function as key signal transducers that channel information via protein phosphorylation/dephosphorylation cycles (Rispail et al., 2009). A MAPK cascade usually contains three kinases ([MAPKKKs (Mitogen-activated protein kinase kinase kinase), MAPKKs (Mitogen-activated protein kinase kinase) and MAPKs]) that are activated sequentially. The activated MAPKs then phosphorylate downstream regulators such as transcription factors, resulting in specific output responses. In fungi, four MAPK cascades have been documented, including the complete Fus3-, Hog1-, and Slt2-MAPK cascades, and the Ime2-MAPK cascade where MAPKKK and MAPKK have not been documented (Xu et al., 2017).

Gene expansion and loss have resulted in significant diversity in the MAPK cascades in the fungal kingdom. A macroevolutionary analysis showed that a complete loss of the MAPK cascades was found in 17 microsporidia, some of which are insect pathogens (Xu et al., 2017). However, in four filamentous insect pathogenic fungi (*Metarhizium, Beauveria, Cordyceps* and *Ophiocordyceps*), Fus3-, Hog1-, and Slt2-MAPK cascades are all identified (Zheng et al., 2011; Xu et al., 2017). In another widely investigated insect pathogenic fungus *Beauveria bassiana*, duplication of the MAPK genes occurred (Tong and Feng, 2019). In contrast, a genome analysis identified complete Fus3-, Hog1-, and Slt2-MAPK cascades in nine *Metarhizium* species including the early diverging species *M. album* and *M. rileyi*, and no gene duplication and loss occurred in these species (Table 1).

**Table 1:** Summary of the MAPK cascade components in the *Metarhizium* fungi.

**Protein names**	**Genbank accession number**							
	***Macs***	***Mro***	***Mbr***	***Mal***	***Mma***	***Mgu***	***Mri***	***Mhu***
**The Fus3 cascade**								
Ste11-MAPKKK	MAC_05029	MAA_03529	MBR_07623	MAM_01551	MAJ_03534	MGU_03913	NOR_03561	MHUMG1_05456
Ste7-MAPKK	MAC_02314	MAA_04421	MBR_08370	MAM_05683	MAJ_09054	MGU_06941	NOR_07221	MHUMG1_06636
Fus3-MAPK	MAC_00098	MAA_04503	MBR_02902	MAM_00092	MAJ_02296	MGU_00109	NOR_01465	MHUMG1_03383
**The Hog1 cascade**								
Ssk2-MAPKKK	MAC_03196	MAA_04290	MBR_03252	MAM_02047	MAJ_00855	MGU_02943	NOR_04771	MHUMG1_06778
Pbs2-MAPKK	MAC_04869	MAA_00856	MBR_05786	MAM_02108	MAJ_02747	MGU_03339	NOR_02670	MHUMG1_04052
Hog1-MAPK	MAC_08084	MAA_05126	MBR_00253	MAM_00548	MAJ_06162	MGU_08121	NOR_02326	MHUMG1_04940
**The Slt2 cascade**								
Bck1-MAPKKK	MAC_04640	MAA_04583	MBR_02986	MAM_00019	MAJ_05883	MGU_00026	NOR_01542	MHUMG1_03301
Mkk1-MAPKK	MAC_07479	MAA_05913	MBR_01250	MAM_02337	MAJ_01232	MGU_01925	NOR_06840	MHUMG1_06089
Slt2-MAPK	MAC_08024	MAA_03181	MBR_04046	MAM_08258	MAJ_08054	MGU_06607	NOR_06270	MHUMG1_09677
**The Ime2 cascade**	MAC_01790	MAA_05403	MBR_01778	MAM_04122	MAJ_03070	MGU_02459	NOR_03976	MHUMG1_01737

*Note: Mac, Metarhizium acridum CQMa 102; Mro, Metarhizium robertsii ARSEF23; Mbr, Metarhizium brunneum ARSEF 3297; Mal, Metarhizium album ARSEF1941; Mma, Metarhizium majus ARSEF 297; Mgu, Metarhizium guizhouense ARSEF 977; Mri, Metarhizium rileyi RCEF 4871; Mhu, Metarhizium humberi*.

## Genetic Characterization of MAPK Cascades in *Metarhizium* Fungi

All 10 components in the MAPK cascades have been systematically characterized in *M. robertsii* (Chen et al., 2016), and a few components in the cascades were also investigated in *M. acridum* and *M. rileyi* (Jin et al., 2012, 2014; Song et al., 2016; Wang et al., 2018). No obvious phenotypic changes were found to result from the deletion of the gene encoding Ime2-MAPK in *M. robertsii* (Chen et al., 2016). In *M. robertsii*, the Fus3-MAPK cascade regulates conidiation, and the three gene deletion mutants in this cascade all showed reduced conidial yields and impaired conidial pigmentation. The Fus3-MAPK cascade is indispensable for cuticle penetration, and the mutants cannot produce appressoria on the insect cuticle. However, this cascade is not involved in hemocoel colonization. The Fus3-MAPK cascade is also indispensable for tolerance to the hyperosmotic stress because the hyphal tips of the mutants swollen and burst under this stress. The Fus3-MAPK cascade is also involved in the tolerance to cell wall disturbing agent Congo Red (Chen et al., 2016). The Fus3-MAPK cascade is generally functionally conserved in the *Metarhizium* genus, but diversifications have also occurred. As with *M. robertsii*, the Fus3-MAPK (MaMK1) is also necessary for appressorium formation and following cuticle penetration, and is not involved in hemocoel colonization (Jin et al., 2014). In contrast to *M. robertsii*, the Fus3-MAPK is not involved in conidiation in *M. acridum* (Jin et al., 2014). In addition, the Fus3-MAPK is necessary for *M. acridum* to reemerge from the dead insects, which however is not found in *M. robertsii* (Jin et al., 2014; Chen et al., 2016).

The Hog1-MAPK is important for tolerance to hyperosmolarity in *M. robertsii, M. acridum* and *M. rileyi*. In contrast to *M. robertsii*, MaHog1 is also important for oxidative stress conferred by H_2_O_2_ in *M. acridum* (Jin et al., 2012). Hog1-MAPK is involved in cuticle penetration and hemocoel colonization in *M. robertsii, M. acridum* and *M. rileyi* (Jin et al., 2012; Chen et al., 2016; Song et al., 2016; Wang et al., 2018). The Hog1-MAPK cascade positively regulates conidiation in *M. robertsii* and *M. rileyi*, and their mutants all showed reduced conidial yields and altered conidial pigmentation (Chen et al., 2016; Song et al., 2016). *In M. robertsii*, the mutant of Hog1-MAPK showed significantly lower conidial yield than the mutants of the MAPKK and MAPKKK in the cascade, which could be due to the cross-talk that occurs between the Slt2-MAPK and Hog1-MAPK cascade during conidiation, with the Slt2-MAPK phosphorylating the Hog1-MAPK (Chen et al., 2016). In *M. robertsii*, the Hog1-MAPK regulates conidiation via control of the central regulatory pathway for conidiation, which mainly contains three sequentially controlled transcription factors (BrlA, AbaA and WetA) and is conserved in the Ascomycete fungi. The *Pks1* gene cluster for the conidial pigmentation is also controlled by the Hog1-MAPK via the central regulatory pathway (Zeng et al., 2017).

So far, the Slt2-MAPK cascade has only been characterized in *M. robertsii*. Slt2-MAPK is involved in cuticle penetration and hemocoel colonization. The Slt2-MAPK lost its ability to produce appressorium, but appressorial formation was only delayed in the mutants of MAPKKK and MAPKK in this cascade. Although the mutant of the Slt2-MAPK cannot produce appressorium, it is still able to infect insects, and this could be due to the upregulation of cuticle-degrading proteases in the mutant, which could facilitate cuticle penetration. As with the Fus3- and Hog1-MAPK cascade, the Slt2-MAPK cascade also positively regulates conidiation; in addition to the reductions in conidial yield and alteration of conidial pigmentation, the colonies of the three mutants in this cascade showed severe sectorization. As describe above, Slt2-MAPK is required for Hog1-MAPK phosphorylation during conidiation. Similar to the Slt2-MAPK cascade in many other fungi, the Slt2-MAPK cascade regulates the tolerance to cell wall disturbing agent Congo Red in *M. robertsii*. This cascade is also involved in tolerance to hyperosmotic stress (Chen et al., 2016).

## The FUS3-MAPK/STE12/AFTF1 Signaling Branch

Comparison of the gene expression of the wild-type (WT) strain with the MAPK mutants using RNA-Seq analysis revealed many genes regulated by the MAPKs in *M. robertsii* (Chen et al., 2016). Further characterization of MAPK-regulated genes has identified several new and important components in the MAPK signaling pathways, which provide significant insights into pathogenesis and conidiation in *M. robertsii* (Guo et al., 2017; Meng et al., 2019, 2021a,b).

During cuticle penetration, the transcription factor AFTF1 with a Zn2Cys6 fungal type DNA binding domain is positively regulated by the Fus3-MAPK cacade. Compared to the WT strain, appressorial formation by the deletion mutant of *Aftf1*, and to a lesser extent the strain *Aftf1*^*OE*^ with *Aftf1* overexpressed was delayed, and their pathogenicity was accordingly reduced. Therefore, a precisely modulated optimal level of the AFTF1 protein is needed to choreograph appressorial formation. The expression of *Aftf1* is induced by the transcription factor STE12 (named as MrSt12 in *M. robertsii*); MrSt12 achieves this by physically interacting with the cis-acting element (ATGAAACA) in the promoter of *Aftf1*. As with the transcription factor STE12 in many other fungi, the activity of MrSt12 is directly phosphorylated by the Fus3-MAPK for activation. In *M. robertsii*, the deletion mutant of *MrSt12* is unbale to form appressorium and is thus nonpathogenic, suggesting that MrSt12 also regulates genes that are not controlled by AFTF1 (Meng et al., 2019). The transcription factor STE12 in *M. acridum* and *M. rileyi* have similar functions, and they regulate appressorium formation as the deletion mutants cannot form mature appressoria (Wei et al., 2017; Lin et al., 2021). Therefore, AFTF1 is a new component in the Fus3-MAPK/STE12 signaling branch that regulates cuticle penetration. Homologs of AFTF1 are found in almost all *Metarhizium* species, and in the plant pathogenic fungi such as *Fusarium oxysporum* (EWY87630) and *M. oryze* (XP_003715433), suggesting that the Fus3-MAPK/STE12/AFTF1 signaling branch could be widespread.

Conversely, the membrane protein Mr-OPY2 negatively regulates *Aftf1* expression to ensure that the expression level of *Aftf1* is not too high for optimum effect (Guo et al., 2017). Upregulation of the Mr-OPY2 protein is required to repress the expression of *Aftf1* and initiate appressorium formation for cuticle penetration, whereas reduced production of Mr-OPY2 elicits saprophytic growth and conidiation. The precise regulation of the Mr-OPY2 protein production is achieved via alternative transcription start sites. During saprophytic growth, only a single long transcript is expressed with small upstream open reading frames in its 5′ untranslated region, which inhibits translation of Mr-OPY2. Increased production of the Mr-OPY2 protein on the insect cuticle is achieved by expression of a transcript variant lacking a small upstream open reading frame, which can be highly translated. In addition to regulation of *Aftf1*, Mr-OPY2 also regulates tolerance to high osmotic stress by controlling the Hog1-MAPK and Fus3-MAPK cascade through the adaptor protein STE50. Mr-OPY2 physically contacts the STE50, which in turn interacts with the MAPKKKs in the Hog1- and Fus3-MAPK cascade. In the yeast *Saccharomyces cerevisiae*, three binding sites (CR-A, CR-B, and CR-D) in the OPY2 protein were found to interact with STE50; CR-A binds to STE50 constitutively to transmit signals to both the Hog1- and Fus3-MAPK cascade, while CR-B binds to STE50 under glucose-rich conditions to transmit the signal preferentially to the Hog1-MAPK cascade (Yamamoto et al., 2010). However, Mr-OPY2 does not have the CR-A, CR-B and CR-D sites, so the how Mr-OPY2 interacts with STE50 in different conditions remains to be determined. Since the high osmotic stress did not upregulate the Mr-OPY2 protein, a low level of this protein may be sufficient for stress resistance. Mr-OPY2 was the first OPY2 protein characterized in the filamentous fungal pathogens, and a recent study on its homolog in the plant pathogenic fungus *Magnaporthe oryzae* showed that the OPY2 protein is generally functionally conserved among the insect and plant pathogenic fungi (Cai et al., 2022).

## The FUS3-MAPK/RNS1 Signaling Branch

Another novel transcription factor RNS1 (regulation of nutrient selection 1) is also directly regulated by the Fus3-MAPK cascade, but shows no direct relationship with the transcription factor AFTF1 (Meng et al., 2021a). RNS1 is a Myb transcription factor containing a SANT domain (pfam00249). On the insect cuticle, the Fus3-MAPK physically contacts and phosphorylates the threonine at position 215 (Thr-215) and the serine at position 226 (Ser-226) in the RNS1 protein, and this facilitates the entry of RNS1 into nuclei. The phosphorylated RNS1 binds to the target DNA motif (ACCAGAC) in its own promoter to self-induce expression. Abundant phosphorylated RNS1 protein activates the expression of the genes for degrading cuticular proteins, chitin, and lipids, and this provides nutrients for hyphal growth and facilitates the entry of the fungus into the insect hemocoel for subsequent colonization (Meng et al., 2021a).

In addition to regulation of hydrolysis of the proteins, chitin, and lipids in the insect cuticle, using the same mechanisms described above on the insect cuticle, the Fus3-MAPK/RNS1 signaling branch also activates the genes for utilizing common complex and less-favored nitrogen and carbon sources (casein, colloid chitin, and hydrocarbons) that are not derived from insects. This is repressed by favored organic carbon and nitrogen nutrients, including glucose and glutamine. Therefore, the Fus3-MAPK/RNS1 branch controls fungal nitrogen and carbon metabolism and is implicated in the entomopathogenicity of *M. robertsii*. Homologs of RNS1 are found in other *Metarhizium* species and many other non-*Metarhizium* Ascomycete fungi including the saprophytes *Neurospora crassa* and *Aspergillus* fungi, and other insect pathogens, plant or mammal pathogens, suggesting that the Fus3-MAPK/RNS1 cascade could be a common mechanism in the phylum of the Ascomycota (Meng et al., 2021a).

Utilization of environmental carbon and nitrogen sources is a key physiological process for fungi to obtain the needed building blocks to sustain life and grow (Luo et al., 2014). Fungi have evolved sophisticated regulatory mechanisms to enable them to respond rapidly to fluctuating availability of carbon and nitrogen nutrients in the environment. In addition to the Fus3-MAPK/RNS1 signaling branch found in *M. robertsii*, two other regulatory mechanisms have been widely documented in fungi, which ensure that the genes for utilization of less-favored carbon or nitrogen sources are expressed only in the absence of the favored nutrients so that fungi preferentially utilize favored carbon (e.g., glucose) and nitrogen (e.g., ammonium and glutamine) sources. One mechanism is the nitrogen metabolite repression (NMR). The regulatory protein NmrA deactivates the GATA transcription factor AREA in the presence of favored nitrogen, whereas in the absence of favored nitrogen sources, NAD^+^ binds to NmrA, which in turn dissociates from AREA, thereby allowing AREA to activate expression of the genes for utilizing less-favored nitrogen sources (Wong et al., 2008). The other mechanism is called the carbon catabolite repression (CCR). Dependent on the repressor CreA, CCR ensures glucose is preferentially utilized by preventing the expression of the genes for utilizing less-favored carbon sources (Beattie et al., 2017). The CCR and NMR are functionally conserved in fungi, including several *Metarhizium* species (Song et al., 2019; Lai et al., 2020; Meng et al., 2021a). However, the Fus3-MAPK/RNS1 signaling branch acts independently from CCR and NMR, and other regulators of fungal carbon and nitrogen metabolism, including Tps1, Snf1, and TOR kinase (Meng et al., 2021a).

## The SLT2-MAPK/RNS1 Signaling Branch

As described above, the *Metarhizium* fungi have the central conidiation regulatory pathway containing the transcription factors BrlA, AbaA and WetA. As with other ascomycete fungi, in *M. robertsii* the *BrlA* gene has two overlapping transcripts *BrlA*α and *BrlA*β; they have the same major ORF, but the 5′ UTR of *BrlA*β is 835 bp longer than the one of *BrlA*α. Multiple upstream ORFs (uORFs) are in the 5′ UTR of *BrlA*β, which could inhibit the translation of the downstream major ORF (Guo et al., 2017; Meng et al., 2021b). During conidiation, the Slt2-MAPK phosphorylates the serine at the position 306 in RNS1. The phosphorylated RNS1 self-induces by binding the BM2 motif (CCCAGAC) in its own promoter, which in turn binds to the BM2 motif in the promoter of the *BrlA* gene and induces the expression of the transcript *BlrA*α. Since no uORFs are in the 5′ UTR of *BrlA*α, the major ORF can be highly translated. The BrlA protein in turn activates the expression of *AbaA* and *WetA* for optimal conidiation (Meng et al., 2021b).

The aerial hyphae of the deletion mutant of *BrlA* cannot differentiate to produce conidiophores (Zeng et al., 2017), whereas the deletion mutant of *Rns1* can produce conidia though its conidial yield is reduced, suggesting that RNS1 only regulates the expression of the transcript *BrlA*α and the transcript *BrlA*β could also function during conidiation. The contributions of *BrlA*α and *BrlA*β to conidiation remain to be clarified. In addition to conidiation, deletion of the gene encoding Slt2-MAPK also reduced the tolerance to hyperosmotic stress and to the cell wall-disturbing agent Congo red; but deletion of *Rns1* had no impact on the tolerance of *M. robertsii* to hyperosmotic stress and cell wall-disturbing agent. Therefore, the Slt2-MAPK cascade has other downstream branches than the Slt2-MAPK/RNS1.

## Conclusion

**Figure 1:**
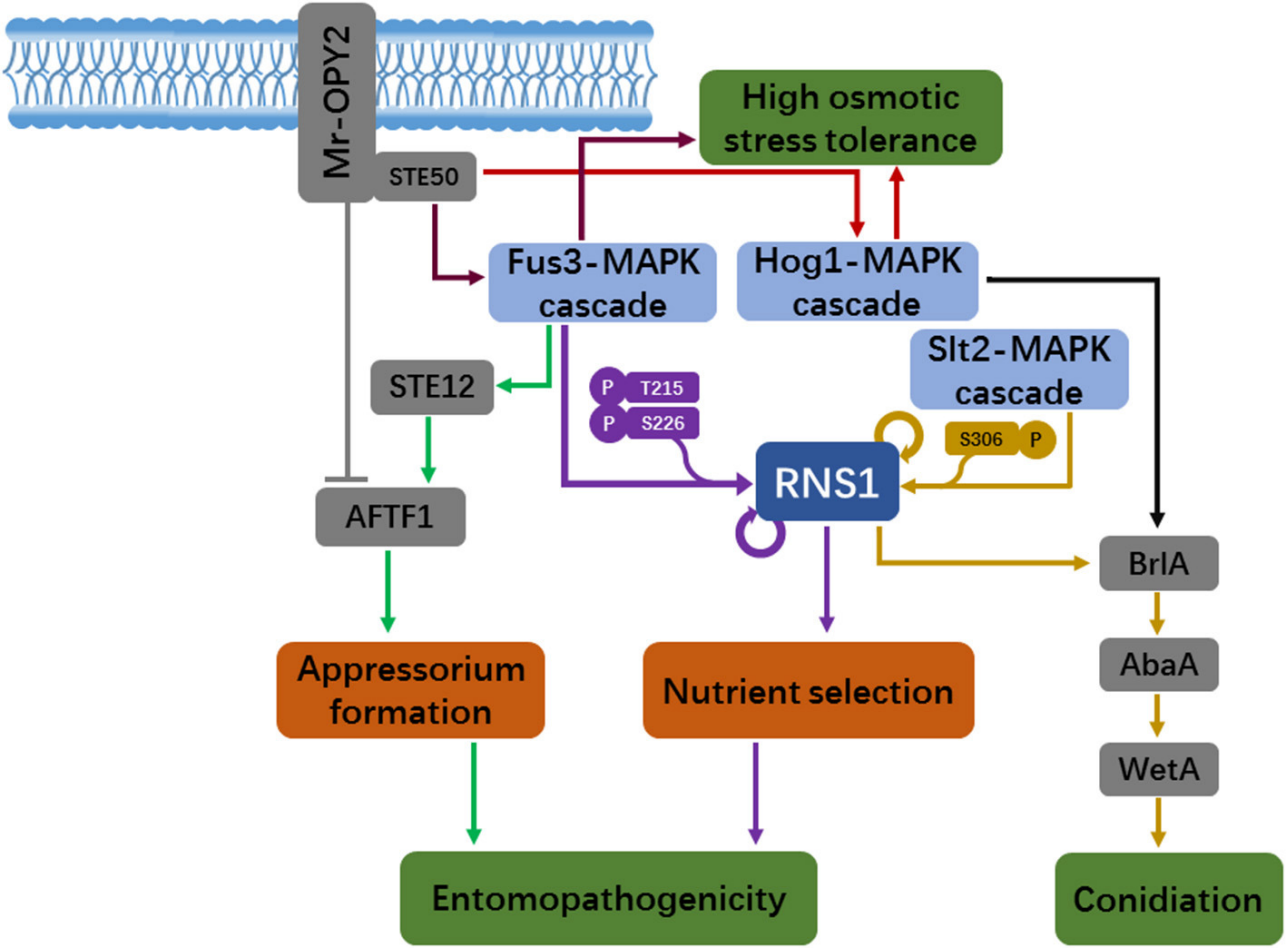
A schematic model of new downstream signaling branches of the MAPK cascades, which regulate asexual reproduction, nutrient selection and insect infection in the fungus *Metarhizium robertsii*. Mr-OPY2-mediated regulation of the MAPK signaling pathways and the transcript factor AFTF1 for appressorial formation and osmotic stress tolerance is also shown. Arrows: induction; Dashes: repression.

The *Metarhizium* fungi have multiple lifestyles and remarkable stress tolerance (Słaba et al., 2013; Moonjely and Bidochka, 2019), resulting in their cosmopolitan distribution. The species *M*. *robertsii* is an emerging model to investigate the mechanisms underlying ecological adaptation in fungi. Major signaling pathways such as the conserved MAPK cascades play key roles in fungal sense and response to fluctuating environmental conditions, which is vital for fungi to persist in environment. In recent years, several novel downstream signaling branches of the MAPK cascades have been discovered in *M. robertsii*, which regulate conidiation and nutrient selection and entomopathogenicity (Figure 1). The Myb transcription factor RNS1 appears to be a central regulator that controls fungal responses to multiple environmental cues. In response to less-favored carbon and nitrogen sources, RNS1 phosphorylated by the Fus3-MAPK activates genes for the utilization of these complex nutrients, which is implicated in entomopathogenicity. The Slt2-MAPK phosphorylates RNS1 during conidiation to activate the central regulatory pathway to initiate asexual reproduction. The transcription factor AFTF1 also appears to act as a hub regulator that channels information from the Fus3-MAPK and the membrane protein Mr-OPY2 for optimal formation of the infection structure appressorium on the host cuticle. As with the components in the MAPK cascades, homologs of RNS1 and AFTF1 are found in other *Metarhizium* species and many non-*Metarhizium* fungi, indicating that these new downstream signaling branches of the MAPK cascades could be widespread.

## Author Contributions

WF conceived the idea and wrote the manuscript. DT and XT wrote the manuscript. All authors contributed to the article and approved the submitted version.

## Funding

This work was supported by the National Natural Science Foundation of China Grants 31872021 and 32172470.

## Conflict of Interest

The authors declare that the research was conducted in the absence of any commercial or financial relationships that could be construed as a potential conflict of interest.

## Publisher's Note

All claims expressed in this article are solely those of the authors and do not necessarily represent those of their affiliated organizations, or those of the publisher, the editors and the reviewers. Any product that may be evaluated in this article, or claim that may be made by its manufacturer, is not guaranteed or endorsed by the publisher.
